# Sensory eye dominance plasticity in the human adult visual cortex

**DOI:** 10.3389/fnins.2023.1250493

**Published:** 2023-08-31

**Authors:** Ka Yee Kam, Dorita H. F. Chang

**Affiliations:** ^1^Department of Psychology, The University of Hong Kong, Pokfulam, Hong Kong SAR, China; ^2^The State Key Laboratory of Brain and Cognitive Sciences, The University of Hong Kong, Pokfulam, Hong Kong SAR, China

**Keywords:** sensory eye dominance, dichoptic perceptual training, perceptual learning, plasticity, fMRI

## Abstract

Sensory eye dominance occurs when the visual cortex weighs one eye’s data more heavily than those of the other. Encouragingly, mechanisms underlying sensory eye dominance in human adults retain a certain degree of plasticity. Notably, perceptual training using dichoptically presented motion signal-noise stimuli has been shown to elicit changes in sensory eye dominance both in visually impaired and normal observers. However, the neural mechanisms underlying these learning-driven improvements are not well understood. Here, we measured changes in fMRI responses before and after a five-day visual training protocol to determine the neuroplastic changes along the visual cascade. Fifty visually normal observers received training on a dichoptic or binocular variant of a signal-in-noise (left–right) motion discrimination task over five consecutive days. We show significant shifts in sensory eye dominance following training, but only for those who received dichoptic training. Pattern analysis of fMRI responses revealed that responses of V1 and hMT+ predicted sensory eye dominance for both groups, but only before training. After dichoptic (but not binocular) visual training, responses of V1 changed significantly, and were no longer able to predict sensory eye dominance. Our data suggest that perceptual training-driven changes in eye dominance are driven by a reweighting of the two eyes’ data in the primary visual cortex. These findings may provide insight into developing region-targeted rehabilitative paradigms for the visually impaired, particularly those with severe binocular imbalance.

## 1. Introduction

Information from the two eyes is not necessarily weighed equally at the level where it is integrated. Such functional asymmetry of the two eyes, thought to originate from the visual cortex is known as *sensory eye dominance* (Coren and Kaplan, 1973). Sensory eye dominance is a prominent characteristic exhibited by some clinical populations, such as patients with strabismic amblyopia (Ding et al., 2013; Zhou et al., 2013), but is also observed in the normal population. Previous studies have found that around 60% of the healthy population shows mild dominance while a significant minority (30–40%) shows strong dominance (Li et al., 2010; Yang et al., 2010; Zhang et al., 2011). Of immediate relevance to the present study, a growing body of work has indicated that mechanisms underlying sensory eye dominance in human adults retain a certain degree of plasticity. In particular, a visual training protocol using dichoptically presented signal-in-noise motion stimuli has gained special traction as it has been demonstrated to effectively reduce eye dominance in both the visually impaired (Hess et al., 2010) and normal observers (Kam and Chang, 2021). To date, much of the research has been focused on developing different paradigms to promote eye-rebalancing (Xu et al., 2010, 2012; To et al., 2011; Li et al., 2013; Tuna et al., 2020), but very little work has been done to reveal the neural underpinnings of sensory eye dominance and its plasticity. Knowledge of such mechanisms may provide insight into how one might develop an efficient rehabilitative protocol.

While the exact neural substrates underlying sensory eye dominance remain unclear, speculative models of how eye imbalance may arrive have been put forth. The two-stage model for binocular integration highlights that the visual system adjusts the relative strength of each eye’s input and integrates these inputs in two distinct stages: The first stage of contrast gain control occurs before binocular combination, where the data from the two eyes remain segregated, but each eye receives inhibitory input from the contralateral eye while the second stage of gain control occurs after binocular combination (Meese et al., 2006). Theoretically, learning-related changes in sensory eye dominance could arise from the changes before, at, or after binocular combination. While this model provides a clear theoretical basis for the modulation and integration of the two eyes’ inputs under normal conditions, the sites of the two posited stages (pre- and post-binocular summation) are still largely unknown. To probe the potential loci of eye balance plasticity, we previously introduced four dichoptic training tasks that differed in terms of the presence of external noise and the visual feature implicated and examined their capacity to drive changes in sensory eye dominance (Kam and Chang, 2021). We found that changes in sensory eye dominance do not depend on the trained task or visual feature, suggesting that the dichoptic training paradigm may at least partially act to balance interocular suppression before or at the site of binocular combination. Therefore, there is reason to believe that dichoptic visual training may act on mechanisms early in the visual cascade, potentially in the lateral geniculate nucleus (LGN) or the primary visual cortex (V1).

The lateral geniculate nucleus is the primary source of feedforward input to V1 (Hubel and Wiesel, 1972; Hendrickson et al., 1978), but it also receives a vast amount of descending feedback from the visual cortex (Van Horn et al., 2000). While LGN neurons are monocular, animal studies have demonstrated that there are interocular inhibitory interactions in the LGN (Sanderson et al., 1971; Marrocco and McClurkin, 1979; Rodieck and Dreher, 1979; Xue et al., 1987; Sengpiel et al., 1995; Dougherty et al., 2021) mediated either by local interthalamic circuits (Guillery and Colonnier, 1970) or corticogeniculate feedback from V1 (Freeman and Tsumoto, 1983; Gaska et al., 2000). The cortico-geniculate feedback projection originating from V1 and (indirectly from) hMT+ is speculated to modulate the strength of the two eyes’ signals before binocular combination (Dougherty et al., 2019b, 2021). It is, therefore, possible for the two eyes to exert different strengths of gain control over signals coming from the other eye at the point where the two eyes’ data are still segregated. While different LGN responses to inputs from one eye or another are not well reported, one fMRI study that used high contrast checkerboard stimuli to assess the functional integrity of the LGN in human amblyopia reported lower LGN activation when driven by inputs from the amblyopic versus the fellow eye (Hess et al., 2009). Moreover, a recent diffusion-weighted imaging study revealed that the white matter microstructural properties of the optic radiations are able to predict the magnitude of sensory eye dominance in the visually normal adults (Chan and Chang, 2022). These findings altogether suggest that mechanisms early in the visual cascade, perhaps the speculative interocular gain control mechanisms at the LGN, may underlie sensory eye dominance in normal-sighted individuals.

Given the current understanding of cortico-geniculate feedback as it relates to modulating the relative strength of the two eyes’ signals, it is reasonable to consider that any changes in the weighting of the two eyes’ data at cortex could result in subsequent changes propagating to the LGN through feedback mechanisms. To our knowledge, however, changes of eye-specific responses in the LGN have only been demonstrated in rodents that have been monocularly deprived for a week (Jaepel et al., 2017). Specifically, the LGN axonal boutons that were initially responsive to the deprived eye exhibited a decrease in their responsiveness to that eye and an accompanying increase in their responsiveness to the non-deprived eye. While there may not be a direct relationship between ocular dominance plasticity and sensory eye dominance plasticity, research on rodents has established a scientifically sound basis for studying sensory eye dominance plasticity within the LGN. A recent functional brain imaging (fMRI) study involving visually healthy human adults attempted to examine whether changes in sensory eye dominance induced by short-term monocular deprivation were accompanied by corresponding changes in the LGN. Their results, however, did not reveal any reliable alterations in LGN activity following 2 h of monocular deprivation (Kurzawski et al., 2022). It is worth noting, however, that the neural mechanisms underlying this kind of short-term plasticity may be different from those induced by a more extended period of monocular deprivation (Ramamurthy and Blaser, 2021) or binocular perceptual training. Here, then, we deem LGN as a particular site of interest to probe changes following dichoptic perceptual training.

We also considered the primary visual cortex as another potential site of interest. From the classic work of Hubel and Wiesel (1968), layer three of V1 is a potential locus of binocular combination as it is the layer within which binocularity emerges. Drawing upon data from more recent work on normal human vision, V1 tended to show an asymmetrical trend of activation during monocular stimulation: higher fMRI signal magnitude during stimulation of the dominant eye and lower magnitude during stimulation of the non-dominant eye, although the dominant eye was determined based on visual and grating acuity (Conner et al., 2007). Studies using visual evoked potential (VEP) (Lunghi et al., 2015a) and fMRI (Binda et al., 2018) have shown that following short-term (2-h) monocular deprivation, activity in V1 increases for the deprived eye but decreases for the non-deprived eye. Further, the activity changes in V1 are correlated with changes in eye dominance. It is unclear, however, as to whether activity changes in V1 might similarly come about after longer-term dichoptic visual training – or whether long-term training results in a different set of neural changes altogether.

The third site we deemed interesting to probe with dichoptic visual training is the human middle temporal complex (hMT+). Based on early neurophysiological studies, the macaque middle temporal area (MT) has been documented to receive retinotopically-organized input from various cortical regions, including V2 and V3 (Maunsell and van Essen, 1983), along with substantial direct input from layers 4B and 6 of V1 (Shipp and Zeki, 1989). It is of also well-established from these classical neurophysiological studies that hMT+ is selective to motion perception (Beckers and Hömberg, 1992; Vaina et al., 2005), including global motion perception (Newsome and Pare, 1988; Braddick et al., 2000, 2001). Considering the functional properties of hMT+ and the fact that the popular dichoptic training task involves a global motion coherence stimulus, hMT+ could then be a possible stimulus-specific locus for the post-summative improvements, although it has not been shown to link specifically to sensory eye dominance plasticity.

Here, using fMRI, we aimed to identify the neural changes associated with improvements in sensory eye balance as driven by dichoptic visual training. We measured changes in blood oxygenated level-dependent (BOLD) activity before and after training in our main sites of interest. We contrasted learning effects following dichoptic signal-in-noise motion training (signal and noise dots presented to different eyes) with training on a binocular variant of the same task (signal and noise dots presented to both eyes). We elected to include a binocular variant of the task for training as it ensures that any differences in learning (and learning associated neural changes) would be due solely to the mode of presentation (dichoptic vs. binocular).

Based on previous work, we predicted that training on the dichoptic, but not binocular variant of the motion signal-noise task would result in behavioral shifts in eye dominance. The rationale behind this is that dichoptic presentation of signal and noise creates viewing conditions that encourage both eyes to work together, whereas binocular presentation of signal and noise does not demand the same level of cooperation between the two eyes, as signal and noise are presented to both eyes equally (i.e., the binocular variant can be solved monocularly). Effective extraction of signal from noise presented to different eyes involves not only a simple summation of the two excitatory monocular inputs but also interocular inhibitory mechanisms, as well documented in the literature (Hess et al., 2007; Zhou et al., 2013). Thus, training under dichoptic, but not binocular presentation, has the potential to promote eye-rebalancing, perhaps by facilitating the combination of the two eyes’ inputs and/or rebalancing inhibitory interactions between the eyes. Further, we reasoned that if perceptual training using dichoptically presented signal-in-noise motion stimuli affects pre-binocular-summation mechanisms, we would observe training-related changes early in the visual cascade (i.e., in the LGN). By contrast, if dichoptic perceptual training acts on mechanisms at or immediately after binocular summation, we would observe learning-associated changes in V1. Lastly, it may well be that dichoptic training results in a reweighing of the signals in higher-order visual mechanisms that are training-feature-specific (in this case, motion-established hMT+, Beckers and Hömberg, 1992; Braddick et al., 2001; Vaina et al., 2005).

## 2. Materials and methods

### 2.1. Participants

Fifty visually normal observers participated in this study (mean age of 22.2 years; SD 3.4 years; 27 males). All had normal or corrected-to-normal visual acuity as screened with the LogMAR chart (0.0) and normal binocular fusion as screened with the Worth-4-dots test. The Worth-4-dots test was performed at 33 cm from the observers, who were shown four dots of light arranged in a diamond configuration (one red dot, two green dots, and one white dot). The observers were required to report the number and color of dots through red/green anaglyph glasses. Normal binocular fusion was indicated by a report of four dots (one red, two green and one mixed color). All the participants were right-handed. They provided written informed consent in line with the ethical review and approval by the Human Research Ethics Committee (HREC), The University of Hong Kong. Participants were randomly assigned to two training groups —receiving training on either a dichoptic (*N* = 25; mean age of 23.6 years; SD 3.7 years; 13 males) or binocular (*N* = 25; mean age of 22.3 years; SD 3.3 years; 14 males) variant of the signal-in-noise motion task. The participants were unaware of their assigned group. One observer from the dichoptic visual training group was excluded from the final analysis due to extensive head movements during the fMRI scan. The sample size was determined based upon statistical power analysis, using the effect size reported in a previous study that employed the same dichoptic training task (Kam and Chang, 2021), with the aim of achieving a minimum of 80% power to detect learning-related changes.

### 2.2. General procedure

**Figure 1:**
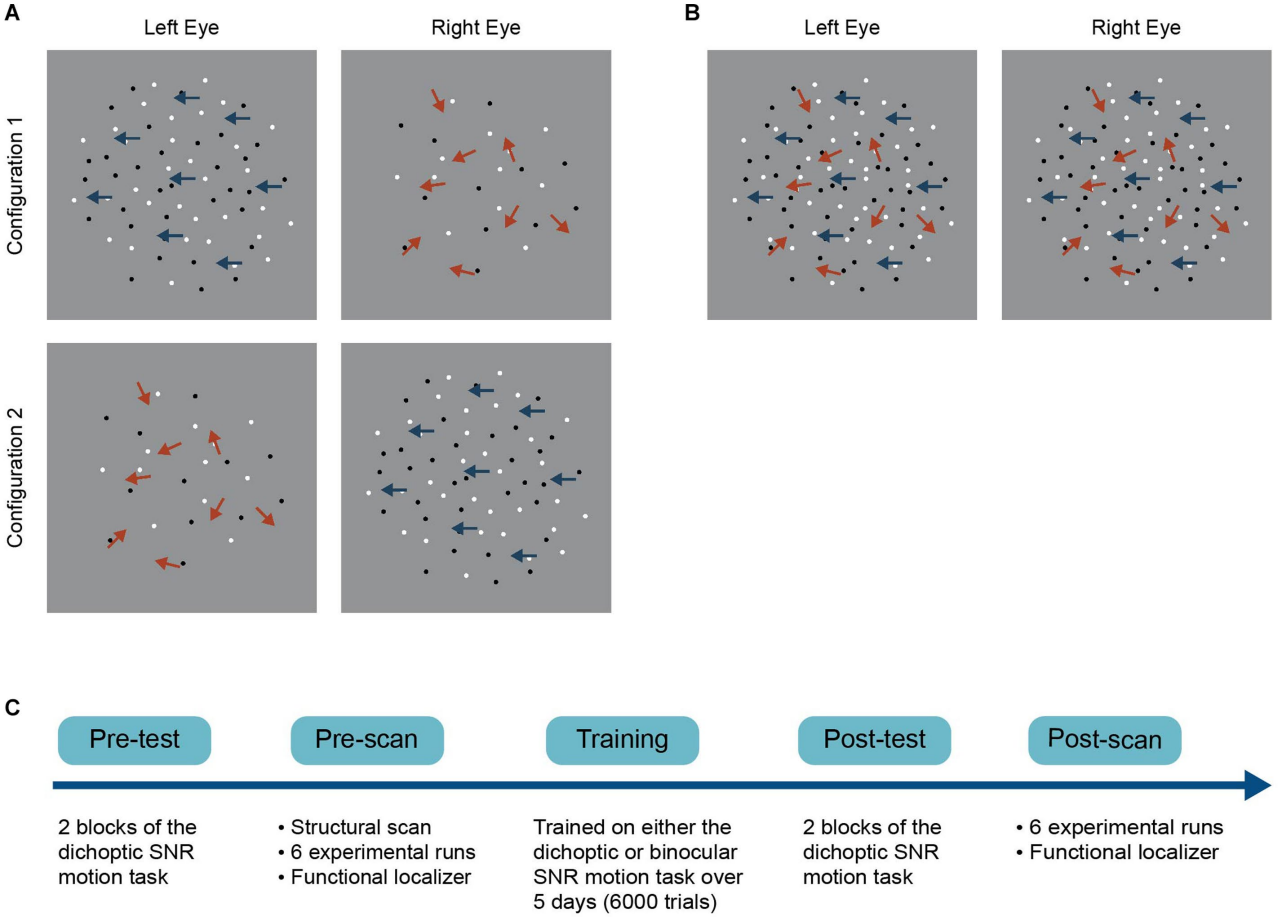
Schematics of the **(A)** dichoptic and **(B)** binocular variant of a signal-in-noise (SNR) motion task and **(C)** the general experimental procedure. For the dichoptic variant, signal and noise dots were presented to different eyes on each trial. Two configurations were used such that we presented signal dots to either the left (configuration 1) or the right eye (configuration 2) on each trial. For the binocular variant, signal and noise dots were presented to both eyes on each trial.

A schematic of the experimental procedure is presented in Figure 1C. Both groups completed pre-training and post-training laboratory tests and fMRI scans. Participants were tested on the dichoptic signal-in-noise (SNR) motion task during the pre- and post-tests. They performed the same task at the pre- and post-scans, during which BOLD signals were measured concurrently. The two groups received training on either the dichoptic or binocular signal-in-noise motion task over five consecutive days (1,200 trials per day, 6,000 trials in total). Each training session lasted 60 min. The post-test was done immediately following the last training session (i.e., after the last training block), while the post-scan was completed the day immediately following the last training session.

### 2.3. In-laboratory testing and training

#### 2.3.1. Apparatus

Stimuli were generated using custom software written in MATLAB, with extensions from Psychtoolbox (Brainard, 1997; Pelli, 1997). Dichoptic presentation of the stimuli was achieved via a shutter-presentation setup that consisted of an ASUS 3D-vision-ready LCD display (resolution: 1920 × 1080 pixels; refresh rate: 120 Hz) paired with NVIDIA 3D Vision 2 shutter glasses. To ensure complete segregation of the two eyes’ signals, we presented different geometric test patterns independently to each eye at the beginning of each session. The observers were instructed to view the geometric test patterns alternatively with their left and right eyes and to report what they observed. No incidents of crosstalk between eyes were reported. The stimuli were viewed at a distance of 50 cm, which was maintained by a chin-rest.

#### 2.3.2. Stimuli and tasks

Stimuli were presented against a uniform gray background and surrounded by a binocularly presented grid-like frame of white and black squares (each 1.5 degrees in size). This grid served to promote binocular fusion by providing an unambiguous background reference. The dichoptic signal-in-noise motion stimuli used in laboratory testing and training were identical to those used in our previous behavioral work (Kam and Chang, 2021). The stimuli consisted of 60 black and 60 white non-overlapping dots presented at 100% contrast. Each dot had a size of 0.2 degrees and moved within a central aperture 9 degrees in diameter (dot density of 1.48 dots/deg^2^) with a velocity of 2 deg/s. The dots did not have a limited lifetime. At the beginning of each trial, the position of each dot was randomly assigned within the aperture. Dots that had an impending collision or were to move outside the aperture on the next update (frame) were redrawn to a random position.

The detectability of motion direction depended on the signal-to-noise ratio, which varied from 0 to 100%. At 100% signal, all the dots moved coherently in either the left or right direction, while at 0% signal, all dots moved in random directions within the aperture. On each trial, we presented signal dots and noise dots dichoptically (i.e., signal and noise dots were presented to different eyes). Observers were asked to make a two-alternative forced-choice judgment of the net motion direction of the dots (either leftward or rightward) by pressing one of two the arrow keys on the keyboard. Task difficulty was manipulated by adjusting the signal-to-noise ratio for each trial using the QUEST staircase procedure, measuring the percentage of signal required to achieve an 82% correctness level. A block of trials (both testing and training block) consisted of two interleaved staircases of 60 trials, with each presenting signal dots to either the left (Figure 1A, configuration 1) or the right eye (Figure 1A, configuration 2). The two staircases were interleaved such that the eye of origin for signals and noise could not be determined on each trial once the two eyes’ images were fused. Stimuli were presented for 500 ms, followed by a 1000 ms response period. Trials were separated by a 300-ms interval. Participants practiced for 20 trials in the pre-test to become familiar with the task and completed two test blocks in both the pre- and post-laboratory tests. Auditory feedback was given during the training (but not the test) sessions to facilitate learning and reinforce correct responses.

All the stimulus and task parameters of the binocular variant of the signal-in-noise motion task were identical to those described above for the dichoptic variant, except that signal and noise dots were presented to both eyes on each trial (Figure 1B). The binocular variant was only used in the training phase (for the binocular training group) and was not tested in the pre- and post-training test.

### 2.4. fMRI acquisition, design, analysis

#### 2.4.1. Apparatus

Stimuli were presented dichoptically in the magnet via a projector (ProPixx, Vpixx) fit with a circular polarizer set to display 1920 × 1080 pixels at 120 Hz. The stimuli were side-projected to a standing mirror placed at 45° behind the bore. The mirror image was then projected onto a translucent 3D rear projection screen placed at the base of the bore. Observers viewed the stimuli through a 45° tilted coil-mounted mirror in front of the head with passive polarized filters inserted in the custom MR-compatible frames. The frames were selected based on the subjects’ intra-pupillary distance. A second optional corrective lens was provided when needed (for myopia). Prior to each scanning session, we verified the segregation of the left and right eye channels by displaying geometric test patterns independently to each eye. Behavioral responses were collected using an MR-compatible response box.

#### 2.4.2. Stimuli and task

Participants were scanned during completion of the dichoptic signal-in-noise motion task. The in-bore stimuli were the same as those used in the laboratory except for the following differences: First, the stimuli were presented at 70% contrast. Second, the size of the central aperture was set to 10  degrees in diameter, with each dot subtending 0.22 degrees and moving with a velocity of 4 deg/s. This change was made to exaggerate the stimulus while minimizing cross-talk in-bore.

#### 2.4.3. fMRI acquisition

Imaging data were acquired using a GE SIGNA Premier 3.0 T scanner with a phased array 48-channel head coil. For both experimental runs and functional localizers, blood oxygen level-dependent signals were measured with a multiband echo-planar sequence (voxel size = 2 × 2 × 2 mm^3^; TR = 2000 ms; TE = 30 ms; FOV = 240 × 240; flip angle = 90°; 58 slices; multiband factor = 2; 200 volumes). Additionally, a high-resolution T1-weighted image was acquired for each participant (voxel size = 1 × 1 × 1 mm^3^; TR = 7 ms; TE = 2.8 ms; FOV = 256 × 256, flip angle = 8°).

#### 2.4.4. Region of interest (ROI) localization

V1 and hMT+ were defined using separate functional localizer scans. We identified V1 using standard phase-encoded retinotopic mapping procedures that mapped polar angles with a slowly rotating checkerboard wedge stimulus (Sereno et al., 1995). Each participant completed clockwise and counterclockwise rotating retinotopic localizer scans. hMT+ was localized using a single-run functional localizer and defined as a cluster of contiguous voxels that showed significantly stronger activation to an array of coherently contracting or expanding dots than to an array of stationary dots (Huk et al., 2002). The LGN was anatomically defined as a 3 mm radius spherical ROI centered on the Talairach coordinate of [left: −22, −24, −2; right: 22, −24 −2] (Chang et al., 2016). The mean Talairach coordinates of V1 and hMT+ as identified in this study, for each hemisphere, are presented in Table 1. The Talairach coordinates of both regions were in good agreement with those reported previously (Poghosyan and Ioannides, 2007; Gaglianese et al., 2012; Aedo-Jury et al., 2020).

**Table 1:** Talairach coordinates (mean ± SD) of V1 and hMT+.

	Left			Right		
	X	Y	Z	X	Y	Z
V1	−8.98 ± 3.82	−89.90 ± 1.97	−5.33 ± 4.45	11.12 ± 2.69	−89.42 ± 1.91	−0.83 ± 4.14
hMT+	−41.66 ± 3.52	−65.45 ± 3.54	−0.43 ± 3.45	41.36 ± 2.15	−63.32 ± 3.18	0.44 ± 3.28

#### 2.4.5. Design and procedure

Before commencing image acquisition, each participant completed one behavior-only run of the dichoptic signal-in-noise motion task while laying inside the bore to obtain thresholds used for computing individually tailored stimulus test values for the main experimental runs. This strategy allowed us to match task difficulty across participants and conditions. Similar to the in-lab tests, one run consisted of two interleaved staircases of 60 trials, each corresponding to the two stimulus configurations (signal dots presented to the left or right eye). We averaged the test values in the last 30 trials for each configuration and defined a range of stimuli values of ±1 SD from this mean value. For each trial, we then sampled the signal-to-noise ratio from this range.

We adopted a block design for the fMRI runs, with each block lasting 16 s. Each run comprised three block types: two stimulus configuration blocks (signal presented to the left or right eye) and a fixation block. Each stimulus block consisted of 8 trials. On each trial, the stimulus was presented for 500 ms and was followed by a 1500 ms response period, during which the observers were asked to judge the net motion direction of the dots by pressing buttons on the response box. A fixation block contained a white fixation cross 0.8 degrees in size that was presented at the center of the screen for 16 s. The order of the stimulus configurations was randomized and stimulus blocks were interleaved with fixation blocks. Within a particular run, each stimulus block was repeated six times, yielding 48 repetitions of a particular stimulus configuration and a total run time of 6 min and 40 s. Each participant completed six fMRI acquisition runs in both the pre- and post-scan. A full scan session lasted around 75 min.

#### 2.4.6. fMRI data analysis

Imaging data were processed using BrainVoyager 22.0. The anatomical data of each participant were transformed into Talairach space and used for cortex reconstruction and inflation. For each functional run, the initial two volumes were discarded to eliminate effects of startup magnetization transients in the data. Functional data were preprocessed using slice scan time correction (with cubic-spline interpolation), 3D head motion correction, linear trend removal, and temporal high-pass filtering (three cycles per run). The preprocessed functional images were subsequently aligned to each participant’s anatomical images and transformed into Talairach space (Talairach and Tournoux, 1988).

We examined univariate responses (general linear model, GLM) and multivariate pattern responses (multivoxel pattern analysis, MVPA). The GLM included regressors for the fixation and the two stimulus configurations (signal dots presented to the dominant eye and signal dots presented to the non-dominant eye) and six motion regressors (three translation parameters and three rotation parameters). The averaged time course signals obtained across all voxels in each ROI were then modeled as a linear combination of the different regressors. The regressor coefficients or beta weights of different stimulus configurations were used for contrasts of the two stimulus configurations. Unlike GLM, which considers overall responsivity, MVPA considers pattern-level responses and their uniqueness to the stimulus configurations. MVPA classification analyses were performed using a linear support vector machine (SVM) classifier. Specifically, the fMRI time course signals of all voxels were first converted to z scores and shifted by 4 s (2 TRs). This shift was introduced to account for the hemodynamic response delay (Serences, 2004). For each ROI, the SVM was trained to classify the patterned responses between the two stimulus configurations: signal dots presented to the dominant eye versus signal dots presented to the non-dominant eye. We adopted a leave-one-run-out cross-validation procedure for the SVM. In each ROI, the functional data of one run was used as the validation dataset, while the remaining runs were used as the training dataset. This analysis was repeated 17 times at all possible fine voxel counts between 10 and 800 voxels (stepping on 50 voxels increment), each computing a classification accuracy at the corresponding voxel count. The final pattern size reported here was determined by the smallest pattern size at which accuracies reached asymptotic levels, corresponding to 400 voxels for our dataset. For each ROI, the mean classification accuracies at 400 voxels were tested against the chance level (0.50), as computed by running 1000 SVMs with shuffled labels.

## 3. Results

### 3.1. Behavior

**Figure 2:**
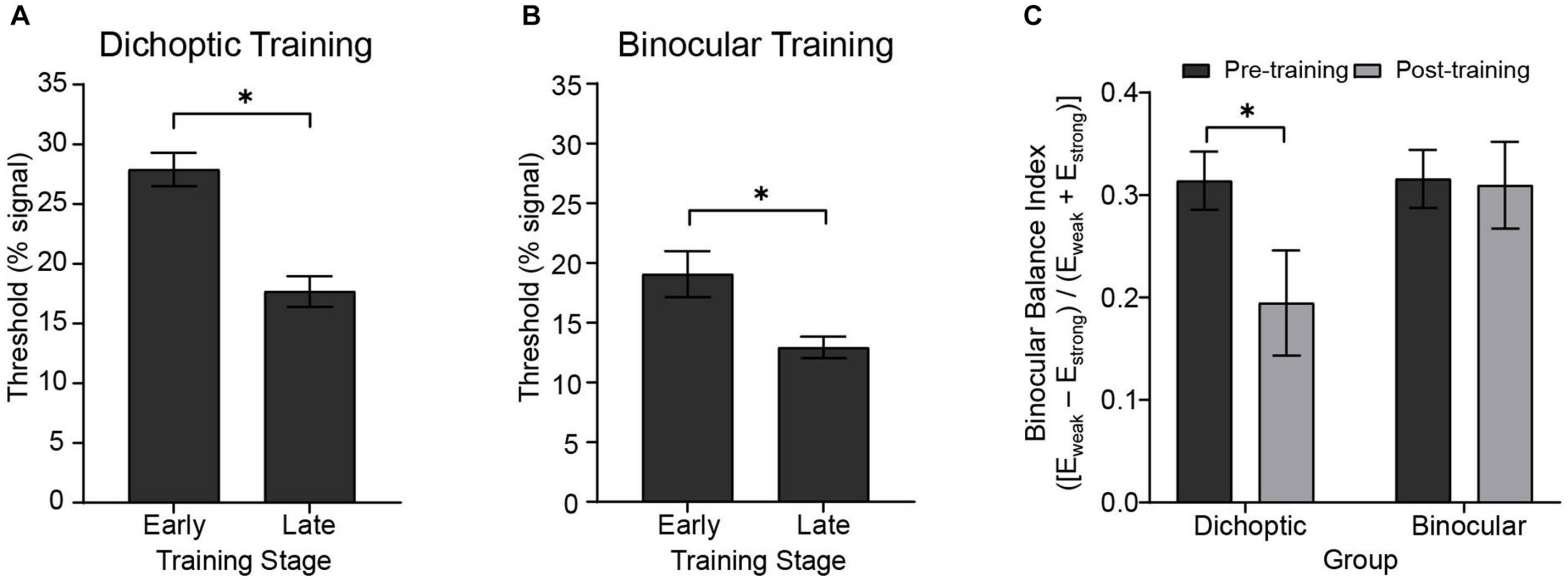
Behavioral results showing the degree of learning and changes in sensory eye dominance. Early and late training thresholds for the **(A)** dichoptic (*N* = 24) and **(B)** binocular (*N* = 25) training groups were derived from averaging the first and the last three training blocks, respectively. **(C)** Sensory eye dominance in the pre- and post-test for the two training groups as indexed by the binocular balance index derived from the dichoptic signal-in-noise motion test task. An index of zero represents no dominance. Error bars represent ±1 SEM, *p < 0.05.

First, we examined the degree of learning achieved by comparing the performance during “early” versus “late” training blocks. For each subject, we averaged motion coherence thresholds (i.e., signal-to-noise ratio) obtained from the first three training blocks and the last three training blocks to represent task performance of the early and late training stages, respectively. We then performed (corrected) paired t-tests independently for the two training groups (Figures 2A,B). The analyses indicated significant improvements in the trained task (i.e., lower thresholds for late vs. early training) for both the dichoptically (*t*_(23)_ = 5.616, *p* < 0.001) and binocularly (*t*_(24)_ = 4.158, *p* < 0.001) trained groups.

We performed two analyses to compare learning effectiveness between training groups. First, we fit individual observer training data with a single-parameter logarithmic function: b=kln(a), where a and b represent the training block and motion threshold, respectively, to determine estimates of the learning rate (k). The learning rate parameter did not significantly differ between the two training groups (*t*_(47)_ = −1.742, *p* = 0.090). Second, we compared threshold changes for the respective training tasks (computed as [(thresh_early training_ – thresh_late training_) / thresh_early training_]). The analysis indicated no significant differences between the two groups in terms of absolute threshold changes (*t*_(47)_ = 0.970, *p* = 0.337).

Next, we examined changes in *sensory eye dominance* attained after the 5-day visual training protocol. As in our previous behavioral work (Kam and Chang, 2021), we quantified sensory eye dominance by deriving a binocular balance index from the dichoptic signal-in-noise motion task. The binocular balance index was computed as (*E*_weak_ – *E*_strong_) / (*E*_weak_ + *E*_strong_), where *E*_weak_ represented the configuration that had a higher threshold (threshold obtained when the signal was presented to the non-dominant eye), and *E*_strong_ represented the configuration that had a lower threshold (threshold obtained when the signal was presented to the dominant eye). An index of zero represented no dominance, and the more the index deviated from zero, the stronger the dominance. For each participant, the dominant eye was identified in the pre-test, therefore yielding a positive binocular balance index in the pre-test. An index closer to zero would indicate a reduction in dominance after training, while any negative value would represent a change of the dominant eye.

**Figure 3:**
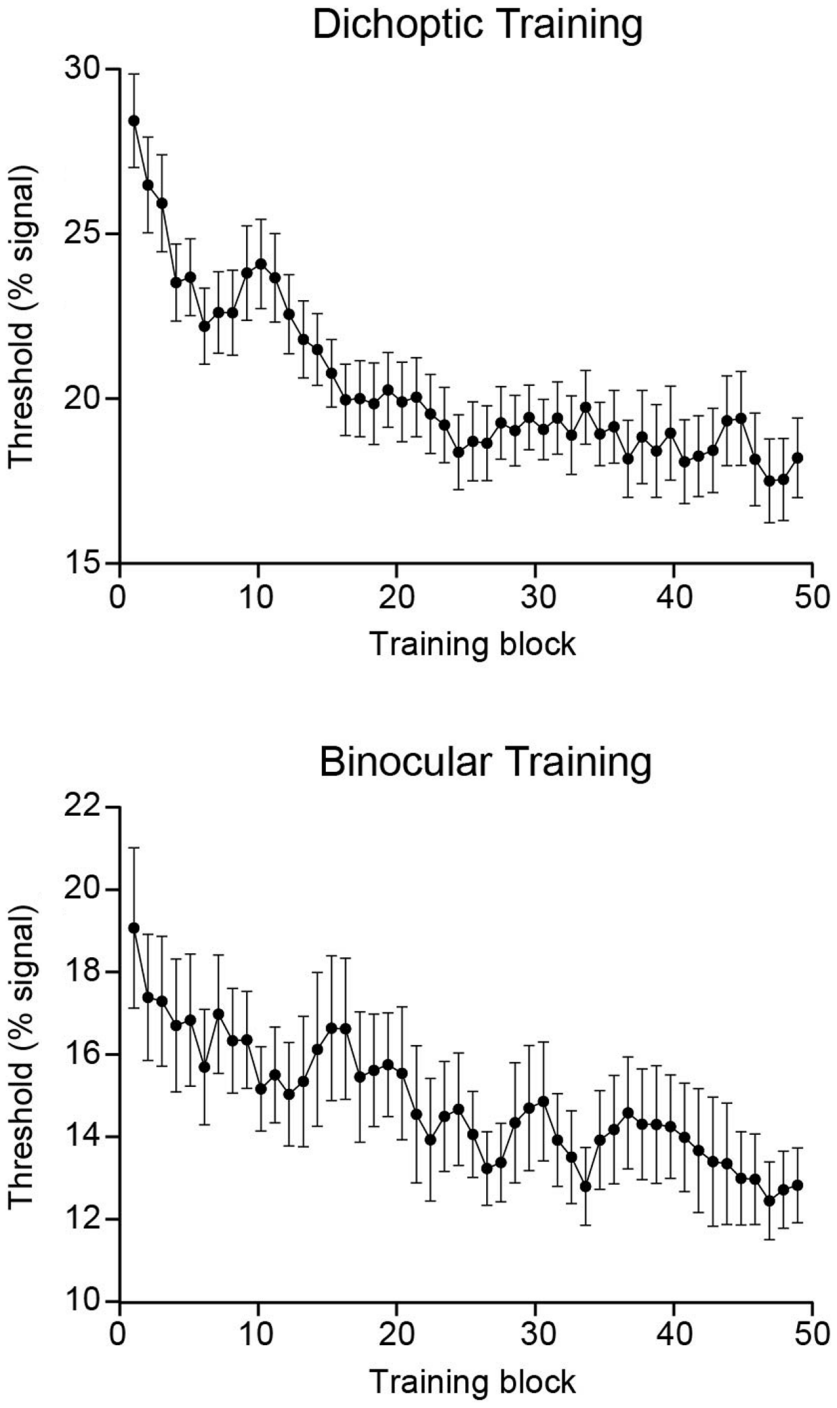
Group-averaged training data presented independently for each training group. Each point represents a three-block moving average. Error bars represent ±1 SEM.

Binocular balance indices were analyzed using a 2 (Group – dichoptic/binocular) × 2 (Time – before/after training) mixed ANOVA that indicated a significant group × time interaction (*F*_(1, 47)_ = 4.13, *p* = 0.048, *n*^2^_p_ = 0.081; Figure 2C). Follow-up paired *t*-tests revealed that only the group that received dichoptic training demonstrated a reduction in the dichoptic motion task-derived binocular balance indices post-training (*t*_(23)_ = 2.66, *p* = 0.014). No change in binocular balance indices was observed in the binocular training group (*t*_(24)_ = 0.185, *p* = 0.855). Notably, the baseline (pre-test) binocular balance index was not significantly different between the two groups (*t*_(47)_ = −0.044, *p* = 0.965). Further, training performance of both groups reached asymptotic levels halfway through the training protocol (~25th block; Figure 3), and learning effectiveness (*k*, above) was comparable between the two groups. Therefore, it is unlikely for the learning-driven changes in sensory eye dominance observed here for the dichoptic-training group to be attributed to dissimilar baseline performances between the two groups or more efficient training for the dichoptically trained group.

### 3.2. fMRI

#### 3.2.1. General linear model (GLM)

**Figure 4:**
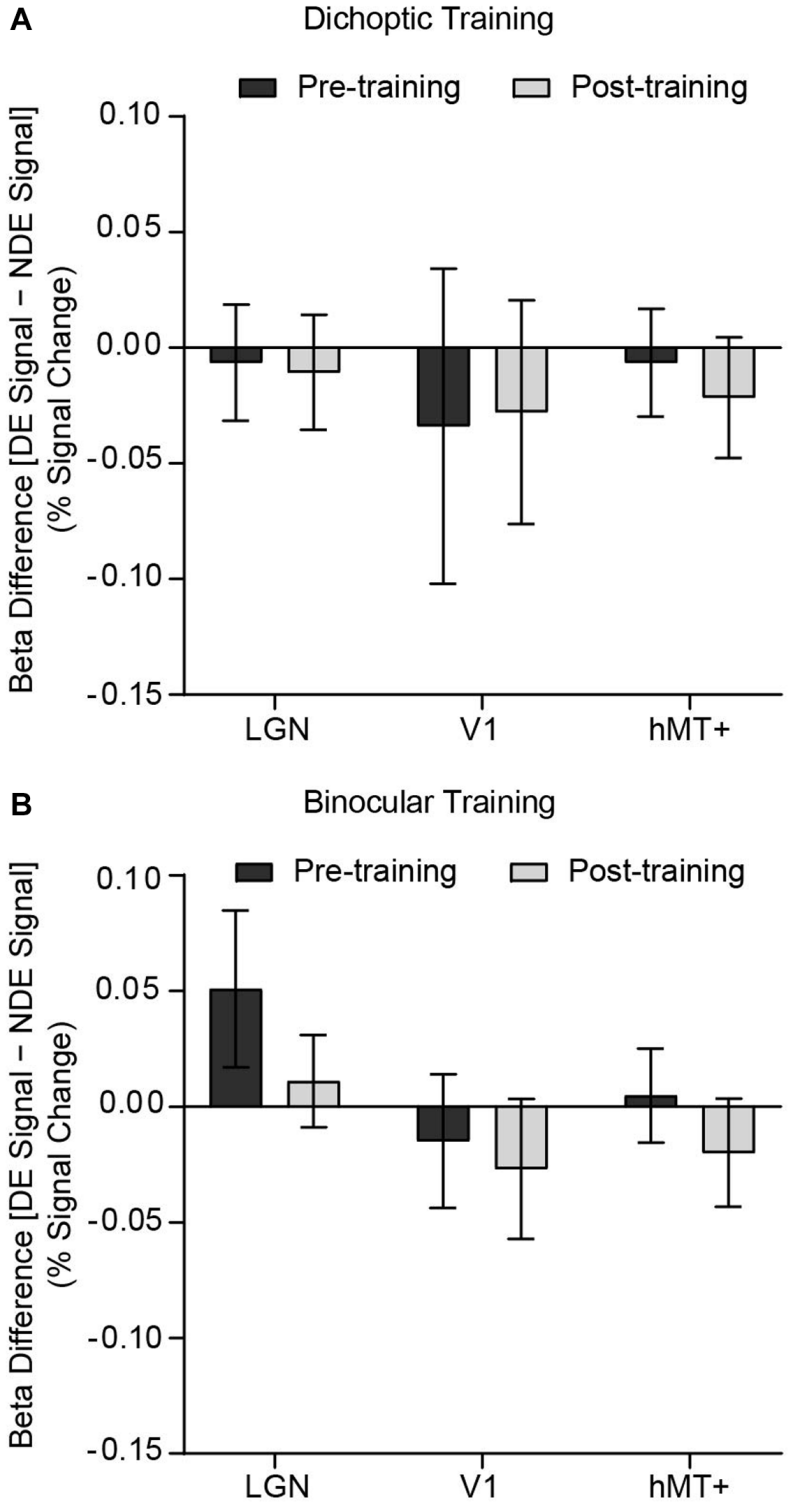
Differences in GLM beta weights [signals presented to the dominant eye (DE signal) – signals presented to the non-dominant eye (NDE signal)] before and after training, presented independently for the **(A)** dichoptic and **(B)** binocular training group. A positive bar represents higher univariate responses when signal dots were presented to the dominant eye. Error bars represent ±1 SEM.

Turning now to the fMRI data, we first evaluated univariate responses in each ROI. For each ROI, GLM beta weights (percent signal change) corresponding to the two stimulus configurations (signal dots presented to the dominant eye and signal dots presented to the non-dominant eye) were extracted for each hemisphere and subsequently entered into a 2 (Group – dichoptic/binocular) × 2 (Time – before/after training) × 2 (Stimulus Configurations – signal dots presented to the dominant eye/signal dots presented to the non-dominant eye) × 2 (Hemisphere – left/right) × 3 (ROI – LGN/V1/hMT+) mixed ANOVA (Figures 4A,B). The analysis revealed a significant main effect of ROI (*F*_(1.48, 69.79)_ = 21.016, *p* < 0.001, *n*^2^_p_ = 0.309) and hemisphere (*F*_(1, 47)_ = 7.336, *p* = 0.009, *n*^2^_p_ = 0.135). Follow-up Bonferroni-corrected comparisons of the beta weights indicated that the LGN (*t*_(48)_ = 3.34, *p* = 0.002) and hMT+ (*t*_(48)_ = 5.74, *p* < 0.001) showed significantly higher responses than V1 (Supplementary Figure 2). In general, signals were stronger in the right hemisphere (*M* = 0.279, SD = 0.407) than the left hemisphere (*M* = 0.184, SD = 0.448; Supplementary Figure 3). Univariate responses did not differ between groups (*F*_(1, 47)_ = 0.903, *p* = 0.347, *n*^2^_p_ = 0.019), time (*F*_(1, 47)_ = 0.179, *p* = 0.674, *n*^2^_p_ = 0.004), and stimulus configurations (*F*_(1, 47)_ = 0.051, *p* = 0.823, *n*^2^_p_ = 0.001). There were no significant interactions. Although the univariate results indicated stronger activations in the right hemisphere compared to the left hemisphere, we elected to concatenate data from the two hemispheres for the subsequent multivariate pattern analyses owing to the fact that univariate amplitudes are removed in multivariate computations.

#### 3.2.2. Multivariate pattern analysis (MVPA)

**Figure 5:**
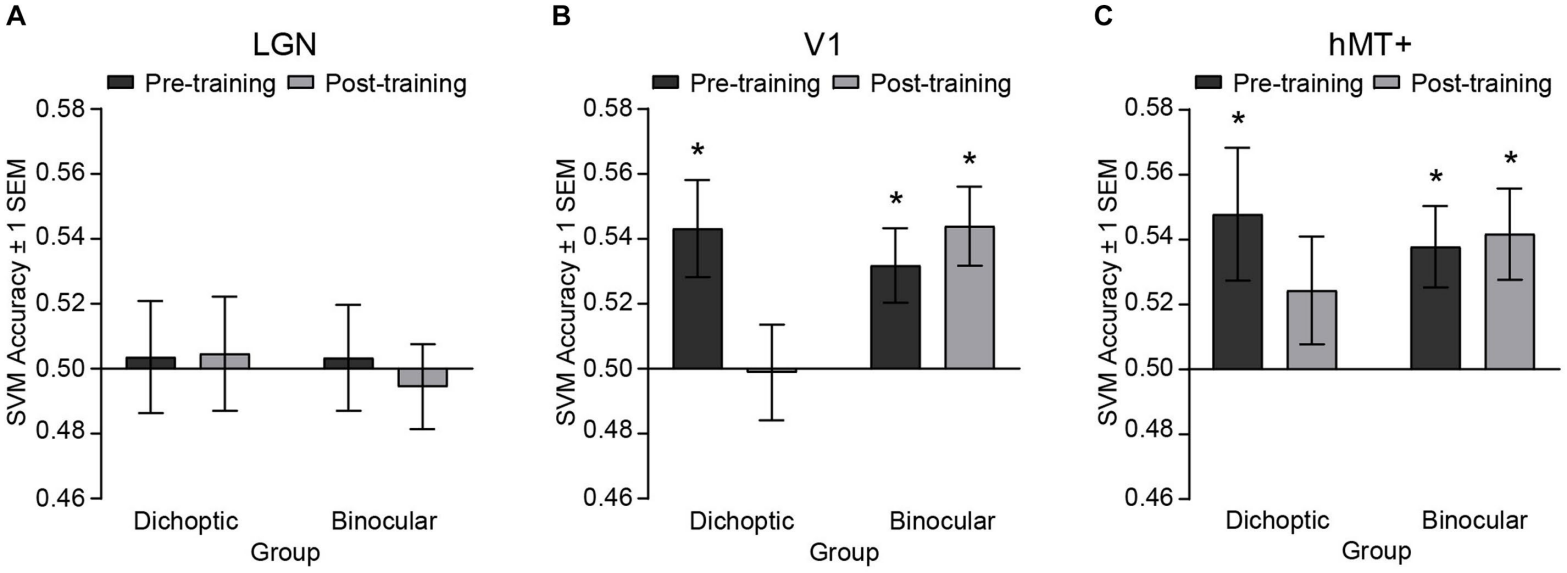
SVM classification accuracies for discriminating fMRI patterned responses between the two stimulus configurations, i.e., [(signal dots presented to the dominant eye) vs. (signal dots presented to the non-dominant eye)] before and after training, presented independently for the **(A)** LGN, **(B)** V1 and **(C)** hMT+. Asterisks denote significant above-baseline (0.5) accuracies. Error bars represent ±1 SEM.

We performed ROI-based multivariate pattern analyses (MVPA) to contrast multivoxel response patterns for the two stimulus configurations [signal dots presented to the dominant eye versus signal dots presented to the non-dominant eye], before and after training (Figure 5). For each (pre and post) dataset and each ROI, classification accuracies were tested against the permutated baseline of 0.50, using *t*-tests while holding false discovery rate (*q* < 0.05).

Before perceptual training, accuracies for classifying between signals presented to the dominant eye versus signal presented to the non-dominant eye were significantly above-baseline in V1 (dichoptic, *t*_(23)_ = 2.89, *p* = 0.008; binocular, *t*_(24)_ = 2.773, *p* = 0.011) and hMT+ (dichoptic, *t*_(23)_ = 2.336, *p* = 0.029; binocular, *t*_(24)_ = 3.007, *p* = 0.006), but not in the LGN (dichoptic, *t*_(23)_ = −0.204, *p* = 0.968; binocular, *t*_(24)_ = −0.586, *p* = 0.564) *for both groups*. After perceptual training, classification accuracy of the LGN remained at chance level for both groups (dichoptic, *t*_(23)_ = 0.263, *p* = 0.795; binocular, *t*_(24)_ = −0.429, *p* = 0.674) but classification accuracy of V1 and hMT+ diverged between the two training groups. For the dichoptic training group, SVM accuracies post-training were no longer above-baseline in V1 (*t*_(23)_ = −0.079, *p* = 0.938) and hMT+ (*t*_(23)_ = 1.462, *p* = 0.157). By contrast, for the binocular training group, SVM accuracies in these two regions remained significantly above-baseline (V1, *t*_(24)_ = 3.604, *p* = 0.001; hMT+, *t*_(24)_ = 2.96, *p* = 0.007). To further compare pre-and post-training SVM accuracies, we conducted separate, corrected 2 (Group – dichoptic/binocular training) × 2 (Time – pre/post) mixed ANOVAs for each ROI. The ANOVAs revealed a significant group × time interaction for V1 only (*F*_(1, 47)_ = 6.24, *p* = 0.016, *n*^2^_p_ = 0.117). Follow-up comparisons indicated that SVM accuracies in V1 were reduced post versus pre-training, specifically for those who received dichoptic perceptual training (*t*_(23)_ = 3.24, *p* = 0.004). No changes in SVM accuracies post versus pre-training were observed in the binocular training group (*t*_(24)_ = −0.68, *p* = 0.503). Overall, the MVPA results indicated that differences in neural responses between the two stimulus configurations within V1 and hMT+ that existed before training were no longer evident after *dichoptic* visual training. In particular, neural responses of V1 changed significantly following *dichoptic* visual training.

Notably, the observed changes in patterned responses after dichoptic perceptual training could not be simply due to the different signal strengths (i.e., signal-to-noise ratio) of the stimuli, or differences in task difficulty, between pre versus post-fMRI scans. The stimuli used for the experimental runs in the bore were individually tailored based on the thresholds obtained from a behavioral run done inside the bore before (pre and post) image acquisition. While the thresholds obtained at the post-scan were lower for both groups, the differences in thresholds between pre and post-scans (thresh_pre_ – thresh_post_) were not significantly different between the two groups (*t*_(47)_ = −0.247, *p* = 0.806). That is, stimulus-level changes were comparable between the two groups, yet changes in the fMRI patterned responses were observed only in those who were *dichoptically* trained. We further verified that our results could not be due to simple differences in task difficulty. To do so, we entered the accuracies of behavioral responses obtained in-bore during the experimental runs into a 2 (Time) × 2 (Group) mixed ANOVA. The analysis revealed no significant effects of group, time, nor interaction, indicating that both groups performed equally well at post-scan versus the pre-scan, even though the signal strength of the stimuli was lower than that of pre-scan. Task performance was also comparable between groups. It is very unlikely, therefore, that the changes in patterned responses after dichoptic perceptual training merely reflect the differences in stimulus difficulty between pre versus post-scans.

Moreover, we verified that the SVM accuracies obtained here were not simply driven by the differences in thresholds between the two stimulus configurations. To test for this, we correlated threshold differences between the two stimulus configurations used in the bore and the SVM accuracies in V1 and hMT+, independently for the pre-training and post-training data. The correlational analyses indicated no significant relationship between the in-bore threshold differences and SVM accuracies in V1 (pre-training, *r*_(47)_ = 0.022, *p* = 0.88, post-training, *r*_(47)_ = −0.121, *p* = 0.407) and hMT+ (pre-training, *r*_(47)_ = 0.07, *p* = 0.633; post-training, *r*_(47)_ = −0.031, *p* = 0.831).

### 3.3. Brain-behavior correlations

As noted above, the patterned responses of V1 and hMT+ were no longer distinguishable between the two stimulus configurations after dichoptic (but not binocular) visual training. In order to better understand the functional relevance of these two brain regions for sensory eye dominance and learning-related changes, we performed an additional set of correlational analyses comparing binocular balance indices and SVM accuracies. Any data points with a Cook’s distance larger than 4/n (where n is the total number of data points) were excluded from the correlational analyses.

**Figure 6:**
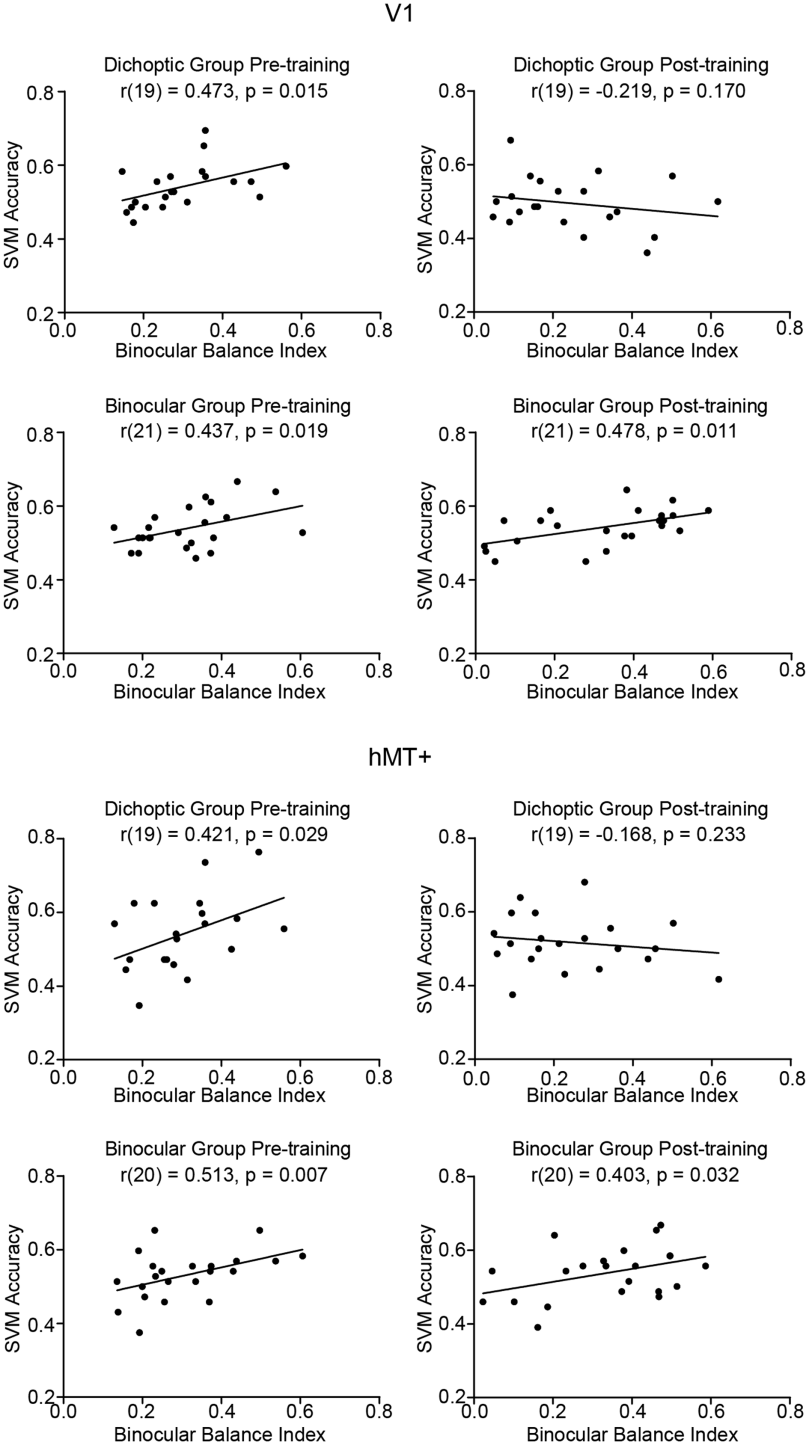
Correlations between SVM accuracies and binocular balance index in V1 and hMT+ before and after training, presented independently for the two training groups. A positive correlation in this context indicates that individuals with stronger eye dominance were associated with a higher pattern-discriminability for the two stimulus configurations in a given region of interest.

We took the absolute value of the post-training binocular balance index such that both pre-and post-training binocular balance indices represented the *degree* of eye dominance. This was done for two reasons: the visual cortex is not concerned with which eye is dominant but rather with the degree of dominance (Muckli et al., 2006). More importantly, SVM accuracy is a non-signed measure that captures only the discriminability of patterned responses between the two stimulus configurations. The dominant eye (left or right) or the change of it is not reflected in SVM accuracy. Therefore, considering only the *degree* of eye dominance (regardless of whether a signage change happened after training) would be more appropriate here. We calculated the correlation coefficient between individual subjects’ SVM accuracies within V1 and hMT+ and the degree of eye dominance, independently for pre- and post-training data (Figure 6). We found that SVM accuracies in V1 (dichoptic, *r*_(19)_ = 0.473, *p* = 0.015; binocular, *r*_(21)_ = 0.437, *p* = 0.019) and hMT+ (dichoptic, *r*_(19)_ = 0.421, *p* = 0.029; binocular, *r*_(20)_ = 0.513, *p* = 0.007) correlated positively to binocular balance index *for both groups*, but only before training. In other words, individuals with stronger eye dominance tended to show higher pattern-discriminability for the two stimulus configurations in these two regions. After training, the positive correlation between SVM accuracies and the degree of eye dominance disappeared for the dichoptic training group (V1, *r*_(19)_ = −0.219, *p* = 0.170; hMT+, *r*_(19)_ = −0.168, *p* = 0.233) but remained for the binocular training group (V1, *r*_(21)_ = 0.478, *p* = 0.011; hMT+, *r*_(20)_ = 0.403, *p* = 0.032). The correlation between post-training hMT+ SVM accuracies and degree of dominance (post-training) for the binocular training group did not survive statistical correction (*q* < 0.05).

Finally, we conducted a separate set of correlational analyses on the post-training data considering only those observers whose post-training binocular balance indices remained unchanged in signage (i.e., no change of the dominant eye). The analyses indicated the same pattern of results as that described above, i.e., SVM accuracy remained positively correlated with the degree of eye dominance only for the binocular training group (V1, *r*_(19)_ = 0.443, *p* = 0.02; hMT+, *r*_(19)_ = 0.366, *p* = 0.05), but not for the dichoptic training group (V1, *r*_(11)_ = −0.134, *p* = 0.317; hMT+, *r*_(11)_ = −0.47, *p* = 0.439).

## 4. Discussion

We examined the neural mechanisms underlying sensory eye dominance plasticity in binocularly normal adults by measuring changes in fMRI responses before and after a five-day visual training protocol. Specifically, we contrasted training-related changes following dichoptic visual training with changes following training on a binocular variant of the same signal-in-noise motion task. We asked whether learning-driven changes in sensory eye dominance were accompanied by alterations in fMRI responses within the LGN, early retinotopic area (V1), and/or stimulus-specific higher-order extrastriate area (hMT+). Whereas both training groups showed improvements on their dedicated training task, shifts in sensory eye dominance were observed only for those dichoptically-trained. The univariate fMRI responses of the three regions of interest showed relatively homogenous activation across stimulus configurations and were largely comparable before and after training. However, results from the pattern level analysis were more revealing. Before training, patterned responses of V1 and hMT+ in both groups were distinguishable between the two stimulus configurations (i.e., whether signal was presented to the strong or weak eye). Interestingly, after dichoptic (but not binocular) visual training, responses of V1 altered and no longer predicted sensory eye dominance.

We consider first, our behavioral findings. In accordance with our previous work, we observed shifts in sensory eye dominance following dichoptic visual training. Instead of comparing the learning effects with a no-training group as we did previously (Kam and Chang, 2021), we included a binocular variant of the same signal-in-noise motion training in the current design. This was done to make the demands on the two groups more comparable, and to verify that any changes in sensory eye dominance after training could not be due to generic motion training. While both variations of signal-in-noise motion training improved performance on the trained task, only the dichoptic training group exhibited shifts in sensory eye dominance following training. The data thus rule out the possibility that learning-driven *eye balance* improvements are attributed to enhancements in motion perception, signal-in-noise extraction, or additional rule-based cognitive enhancements induced by perceptual training, as put forth by previous work involving adults with amblyopia (Zhang et al., 2014).

Notably, upon inspection of the absolute thresholds for the respective training tasks (i.e., dichoptic training thresholds for the dichoptic training group and binocular training thresholds for the binocular training group), the binocular training group had lower thresholds throughout training (Figure 3). Compared to the dichoptic configurations in which ‘signal’ and ‘noise’ dots transit through only one eye, it may not be surprising to have better task performance under binocular configurations, where both signal and noise dots are available to both eyes; hence local motion information (both signal and noise) might be more salient. The higher salience of local motion data could facilitate motion signal integration and assist in determining global motion direction. Despite this difference, we consider the comparison between the two groups to be fair because their learning effectiveness (i.e., the magnitude of learning and learning rate) was comparable, and the baseline (pre-test) threshold on the dichoptic signal-to-noise motion task did not differ significantly between the groups. That is, both groups started at a similar performance level (on the dichoptic test task) and followed a comparable learning trajectory.

We next turn to our fMRI data. Results from the GLM indicated greater overall response amplitudes in the right versus the left hemisphere. This falls in line with previous lesion (Delis et al., 1986; Robertson et al., 1988) and fMRI studies (Martinez et al., 1997; Han et al., 2002) that have shown the right hemisphere to be dominant for processing information that requires global integration of visual stimuli, as would be required for signal integration in our tasks. Further, past behavioral work testing performance on a signal-in-noise motion discrimination task in different visual hemifields reported slightly better task performance when the stimuli were presented to the left versus the right visual field, suggesting a modest right hemisphere dominance for motion signal-in-noise processing in visually healthy individuals (Bosworth and Dobkins, 1999).

Next, we consider the MVPA results. Critically, our multivariate results revealed that V1 and hMT+ are implicated in sensory eye dominance, with the relevance of responses of these two regions to eye dominance disappearing after dichoptic perceptual training. Specifically, before perceptual training, responses of V1 and hMT+ appeared to vary when the signals were presented to different eyes, with the differences in responses escalating with the strength of eye dominance. After dichoptic (but not binocular) visual training, the fMRI patterned responses in V1 and hMT+ no longer predicted sensory eye dominance. However, changes in V1 only survived further stringent statistical evaluation (i.e., ANOVA, group × time interaction). Our findings suggest that dichoptic perceptual training may drive a rebalancing of activity in the primary visual cortex.

V1 is a particularly interesting region to consider in this work, not because of its role as the primary cortico-geniculate receiving area, but because it is where we find the emergence of binocularity. While the MVPA results appear to suggest that V1 reweighs two eyes’ input after dichoptic perceptual training such that it considers the data from the two eyes in a more balanced way, our results do not provide further information about the exact mechanisms or layer-specific changes in V1. Based on the prevailing understanding of the excitatory and inhibitory circuits involved in binocular combination (e.g., Meese et al., 2006), a balanced visual system is maintained, at least in part, by approximately equivalent reciprocal inhibition between the anatomically segregated inputs from each eye to V1. Any asymmetry in interocular inhibition may thus contribute to eye dominance (Sengpiel et al., 1994; Huang et al., 2010). Inhibitory interaction between the eyes is thought to be mediated by the inhibitory gamma-aminobutyric acid (GABA) circuit, as revealed by animal studies (Sengpiel, 2005; Sengpiel et al., 2006; Harauzov et al., 2010). Recently, a study involving normal-sighted human observers found that individuals with stronger eye dominance tended to have a greater interocular difference in GABAergic inhibition in V1, suggesting a different strength of inhibitory influence of one eye over the other eye during active viewing (Ip et al., 2021). The disappearance of the relevance of V1 responses to eye dominance observed in the current study may thus reflect a rebalancing of interocular suppression. Indeed, it has been reported that resting GABA concentration in V1 can be reduced after short-term monocular deprivation, with the degree of reduction correlating with the changes in sensory eye dominance (Lunghi et al., 2015b). Although monocular deprivation and perceptual training may alter sensory eye dominance through different mechanisms, there is reason to speculate that dichoptic perceptual training may act to balance interocular inhibition in V1, presumably by weakening inhibition from the dominant eye, and/or strengthening inhibition from the non-dominant eye.

From the early classic work, it is well documented that information from the two eyes remains largely segregated in the input layer 4 (Hubel and Wiesel, 1968; Blasdel and Fitzpatrick, 1984) and only converges at the layer above (Hubel and Wiesel, 1968). Despite the segregation, a recent study reported (both faciliatory and inhibitory) interocular interactions between monocular neurons in the input layer, before the point where monocular signals integrate (Dougherty et al., 2019a). It is possible, therefore, that the resultant rebalancing effects occur either at the monocular neurons in the input layer before excitatory binocular summation or at the binocular neurons in the upper layers after binocular combination. In this vein then, future work involving ultra-high-field, high-spatial-resolution fMRI may be useful to arrive at learning-driven changes at the laminar level (Olman et al., 2012; Huber et al., 2015; Kashyap et al., 2018).

In addition to V1, our findings reveal that hMT+ is also implicated in sensory eye dominance, at least under the present context. Neurophysiological studies have suggested that MT receives a significant amount of input not only from V1, but also from the monocular projections directly from the koniocellular layers of the LGN (Sincich et al., 2004; Nassi and Callaway, 2006; Warner et al., 2010). Neuroimaging studies imaging effective connectivity (Gaglianese et al., 2012) and tractography (Lanyon et al., 2009) have further demonstrated direct connections between the LGN and hMT+ in the human brain. It is thus not surprising for the responses in hMT+, a stimulus-specific higher-order extrastriate area, to be able to predict sensory eye dominance, as an imbalance between data from the two eyes may already exist in the feedforward input originating directly from the LGN and V1. Further, while neurons in hMT+ are binocular, studies of macaque MT have shown that a substantial proportion of these neurons exhibit ocular preference (Maunsell and Van Essen, 1983; Kiorpes et al., 1996; El-Shamayleh et al., 2010). This means that although the neurons respond to input from either eye, they exhibit varying degrees of preference for input from one eye over the other. This ocular preference, together with the (inherited) imbalanced projections from the LGN and V1, may provide reasonable conduits for the observed relevance of hMT+ to sensory eye dominance before perceptual training.

As previously noted, the results of hMT+ after dichoptic perceptual training are statistically weaker than those of V1, and as such we have been careful not to overstate its relevance here. Following the same logic described above, if the information from the two eyes is reweighted in V1 after dichoptic perceptual training, we would expect hMT+ to exhibit corresponding changes in their responses. However, it is worth reiterating that hMT+ also receives direct monocular projections from the LGN (Lanyon et al., 2009; Gaglianese et al., 2012). Although the LGN-hMT+ pathway is less prominent than the geniculo-striate route (Gaglianese et al., 2012), its existence leads to the speculation that the weaker learning-related changes in hMT+ in this study may be partly attributed to the lack of training effects from the inputs originating from the LGN.

At first glance, our behavioral data appear to be in good consensus with the MVPA results (i.e., reduced behavioral binocular balance index accompanied by reduced pattern-discriminability in V1 and hMT+ after dichoptic perceptual training). Upon close examination, one may notice that while the binocular balance index of the dichoptic training group improved (decreased) with training, it did not drop to zero; however, the residual eye dominance is not reflected in the post-training fMRI data. This raises an intriguing question of where the residual eye dominance is represented. A possible answer for this might be related to something we did not image here: white matter. Microstructural abnormalities (i.e., reduced structural integrity and greater diffusivity) in the optic radiations have been reported in adults with amblyopia (Allen et al., 2015; Duan et al., 2015). Similarly, the aforementioned diffusion-weighted imaging work also found white matter diffusivity in the optic radiations to predict the degree of sensory eye dominance in visually-normal adults (Chan and Chang, 2022). Therefore, it may be the case that sensory eye dominance is reflected in both gray and white matter. Any residual eye dominance, then, could originate from white matter structural differences that could not be targeted by dichoptic training. While our current data do not permit us to determine whether learning induced any alterations in the white matter, it is important to note that the plasticity of gray and white matter could involve very different cellular and molecular mechanisms (Zatorre et al., 2012). Evidence of visual-training-induced microscopic changes within the white matter is scarce. However, data from other domains, such as the motor (Scholz et al., 2009) and cognitive (Takeuchi et al., 2010; Mackey et al., 2012; Schlegel et al., 2012) work, seem to suggest that longer-term training (at least 6 weeks) is required to drive changes in white matter properties. It is, therefore, possible that the same training paradigm (including its duration) may not be equally effective in driving changes in both gray and white matter. For that reason, we speculate that the white matter continues to play a part in eye dominance after training.

To further complicate the story, it is important to consider the fact that LGN receives about 90% of its input from sites other than the retina (Sherman and Guillery, 2002), including a majority of the input originating from V1 (Van Horn et al., 2000). In principle, then, changes in V1 can in turn feedback to influence LGN responses. Our data provided no indications of learning-related changes in the LGN, however. Compared with the visual cortex, evidence of sensory eye dominance plasticity in human subcortical regions is considerably more limited. To our knowledge, the only study to date that has examined eye balance plasticity in the human LGN also failed to detect any changes in BOLD activity after short-term monocular deprivation (Kurzawski et al., 2022). The lack of LGN-related-effects here, and in work of others could reflect a true absence of a role for this region in governing eye balance. Alternatively, it is important to consider several challenges that the LGN poses.

First, the LGN is much smaller in size (and hence weaker signal-to-noise ratio) as compared to V1 and hMT+. As most of our participants had relatively balanced eyes (even before training), response amplitude differences between stimulus configurations or after learning in the LGN may be more difficult to detect under these circumstances. Another possible explanation for our results is that dichoptic perceptual training may drive changes in subcortical and cortical visual processing via mechanisms that are very different in nature. The LGN and the visual cortex (V1 and hMT+) are fundamentally different in a way that most of the cells in the LGN are monocular (Casagrande and Boyd, 1996), while those in V1 (Hubel and Wiesel, 1968) and hMT+ (Maunsell and Van Essen, 1983) are mainly binocular. Also, interocular modulations in the LGN are predominately inhibitory or suppressive (Dougherty et al., 2021), but those in V1 can be facilitatory (Dougherty et al., 2019a). Indeed, various rodent studies have indicated that the characteristics of changes observed in the LGN and visual cortex following monocular deprivation are very different. For instance, a study in which miniature excitatory postsynaptic currents (mEPSC) in adult mice were recorded showed that after monocular deprivation, the mEPSC amplitude of deprived-eye neurons increased in both the LGN and the visual cortex (Krahe and Guido, 2011). However, the increased mEPSC amplitude in the LGN was also accompanied by an increase in frequency (Desai et al., 2002), suggesting that experience-dependent plasticity in the LGN and visual cortex could be mediated by different presynaptic and/or postsynaptic mechanisms. It is also worth noting that many of these rodent studies that have detected eye-specific response changes in the LGN mainly used invasive neurophysiological techniques that tracked individual neurons (Desai et al., 2002; Krahe and Guido, 2011; Jaepel et al., 2017), in contrast to the non-invasive but indirect method of fMRI here. It is therefore possible that there are changes in the LGN driven by dichoptic perceptual training here that are not well reflected by BOLD activity.

We close by highlighting two outstanding issues that merit empirical attention in future research. The first issue pertains to the pattern of results that we would observe in individuals with binocular visual impairments, particularly those with amblyopia. Amblyopia is a neurodevelopmental disorder that arises from prolonged abnormal visual experiences during the critical period of visual development (McKee et al., 2003). This may result from ocular misalignment (strabismus) or unequal refractive power (anisometropia). The model proposed for binocular interactions in amblyopia shares a similar physiological framework with that of normal vision, with the exception of an attenuated signal for the amblyopic eye and an exaggerated imbalance of interocular suppressive signals before binocular combination (Mansouri et al., 2008). If the primary difference in binocular interactions between individuals with normal vision and those with amblyopia is the strength of imbalance in interocular inhibition, it is possible that dichoptic perceptual training could bring about similar changes in the amblyopic brain as those in the visually-normal brain (i.e., a reweighting of the input from the two eyes). However, such changes might be observed at multiple locations along the visual cascade, as individuals with amblyopia exhibit asymmetric activations for the amblyopic eye and the fellow eye not only in V1 (Li et al., 2007), but also in the LGN (Hess et al., 2009) and in extrastriate visual areas (Muckli et al., 2006). It is also important to consider that the training regimen employed in this study could be inadequate in driving changes in sensory eye dominance in amblyopia, as a previous study that trained adult amblyopes using a noise-based dichoptic motion training paradigm reported that a considerably greater number of trials, ranging from 18,000 to 120,000, was required to attain balanced performance (Hess et al., 2010). As such, future studies may need to design a training paradigm that is specifically tailored to this population.

The second issue relates to the durability of training-related neural changes. In our previous behavioral work, we retested three observers after their original post-training test and found that their behavioral changes in sensory eye balance were retained for at least 20 weeks (Kam and Chang, 2021). Although no follow-up fMRI data were collected in the current study, we speculate that if the behavioral changes were sustained for 20 weeks, neural changes may have similarly endured over that period. Nonetheless, further longitudinal investigations are necessary to determine the exact duration of the (behavioral and neural) changes.

Combining behavioral training and fMRI paradigms, we show for the first time how dichoptic perceptual training drives sensory eye dominance plasticity in the visual cortex. Our data suggest that visual training using dichoptic presentation of signal-in-noise motion stimuli leads to changes in sensory eye dominance by potentially driving a reweighting of data from the two eyes in the primary visual cortex. Our findings establish a foundational basis for future work that seeks to better understand training-related improvements at cortex – for example, the interplay between cortical inhibition and excitation. Future research could also explore sensory eye dominance plasticity within the white matter. Reaching a better understanding of binocular plasticity and the neural underpinnings of eye dominance is essential for developing effective rehabilitative paradigms for the visually impaired.

## Data availability statement

The original contributions presented in the study are included in the article/Supplementary material, further inquiries can be directed to the corresponding author.

## Ethics statement

The studies involving humans were approved by Human Research Ethics Committee (HREC), The University of Hong Kong. The studies were conducted in accordance with the local legislation and institutional requirements. The participants provided their written informed consent to participate in this study.

## Author contributions

KK: data curation, formal analysis, writing—original draft, and writing—review and editing. DC: conceptualization, funding acquisition, investigation, methodology, and writing—review and editing. All authors contributed to the article and approved the submitted version.

## Funding

This work was supported by a General Research Fund (17612920) from the University Grants Committee (Hong Kong) to DC, and a grant from the Key-Area Research and Development Program of Guangdong Province (2019006) to DC.

## Conflict of interest

The authors declare that the research was conducted in the absence of any commercial or financial relationships that could be construed as a potential conflict of interest.

## Publisher’s note

All claims expressed in this article are solely those of the authors and do not necessarily represent those of their affiliated organizations, or those of the publisher, the editors and the reviewers. Any product that may be evaluated in this article, or claim that may be made by its manufacturer, is not guaranteed or endorsed by the publisher.
