# Supplementary material for: Sensory eye dominance plasticity in the human adult visual cortex
Source: Front Neurosci. 2023 Aug 31;17:1250493. doi: 10.3389/fnins.2023.1250493 (PMC10513037; doi:10.3389/fnins.2023.1250493)
Supplement: Supplementary file 1 [file Data_Sheet_1.PDF]

Supplementary Material

Sensory Eye Dominance Plasticity in the Human Adult Visual Cortex

Ka Yee Kam, Dorita H. F. Chang*

*** Correspondence:** Dorita H. F. Chang: changd@hku.hk

1 Supplementary Figures and Tables

1.1 Supplementary Figures

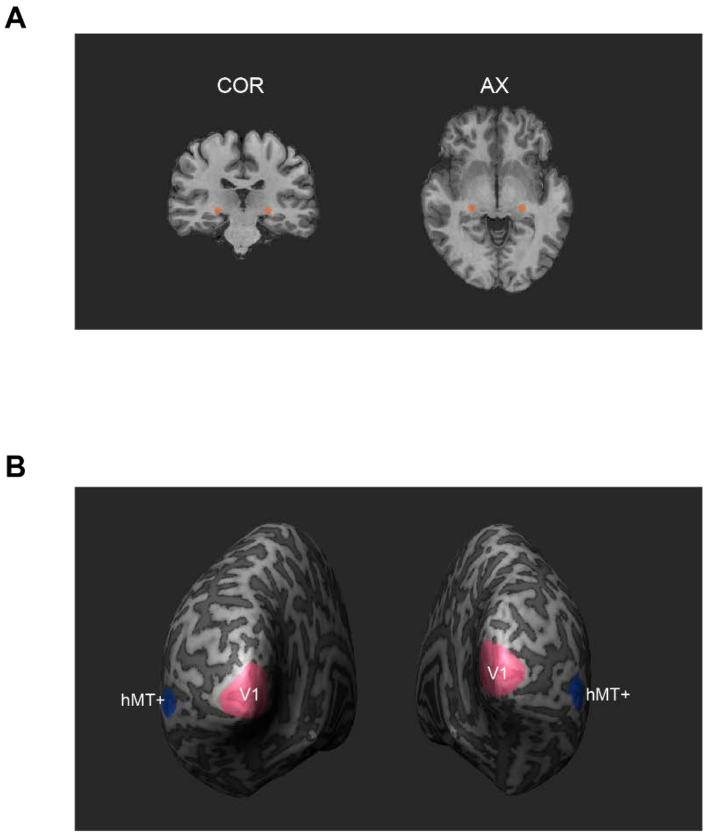

Supplementary Figure 1. An example of the localization of the (A) LGN, (B) V1, and hMT+ in one participant. The LGN was anatomically defined as a spherical ROI, with a radius of 3mm, centered on the Talairach coordinate of [left: -22, -24, -2; right: 22, -24 -2] (Chang et al., 2016). V1 and hMT+ were defined using separate functional localizer scans. V1 was localized using standard phase-encoded retinotopic mapping procedures that mapped polar angles with a slowly rotating checkerboard wedge stimulus (Sereno et al., 1995). hMT+ was defined as a cluster of contiguous voxels that showed significantly stronger activation to an array of coherently contracting or expanding dots than to an array of static dots (Huk et al., 2002).

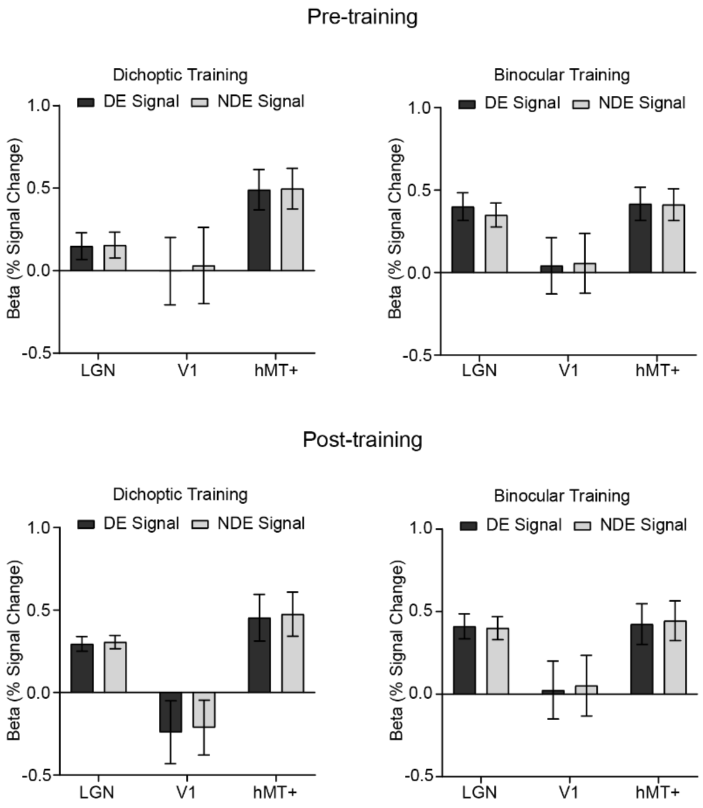

Supplementary Figure 2. GLM beta weights (% signal change) of the two stimulus configurations, i.e. [signal dots presented to the dominant eye (DE signal) and signal dots presented to the non-dominant eye (NDE signal)] before and after training, presented independently for the two training groups. Univariate responses were generally higher in the LGN and hMT+. Error bars represent ±1 SEM.

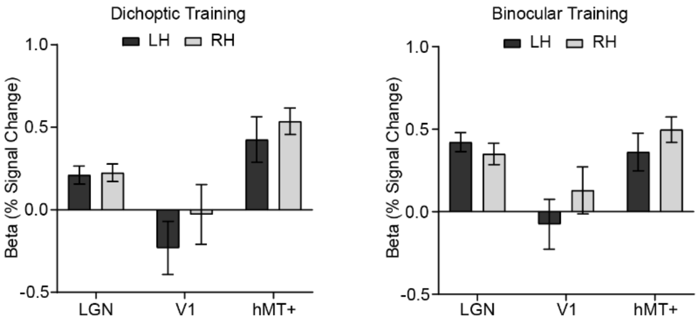

Supplementary Figure 3. GLM beta weights for the left and right hemispheres, presented independently for the two training groups. GLM beta weights for the two hemispheres were collapsed from the two stimulus configurations before and after training. Univariate signals were globally higher in the right hemisphere (RH) than in the left hemisphere (LH). Error bars represent ±1 SEM.

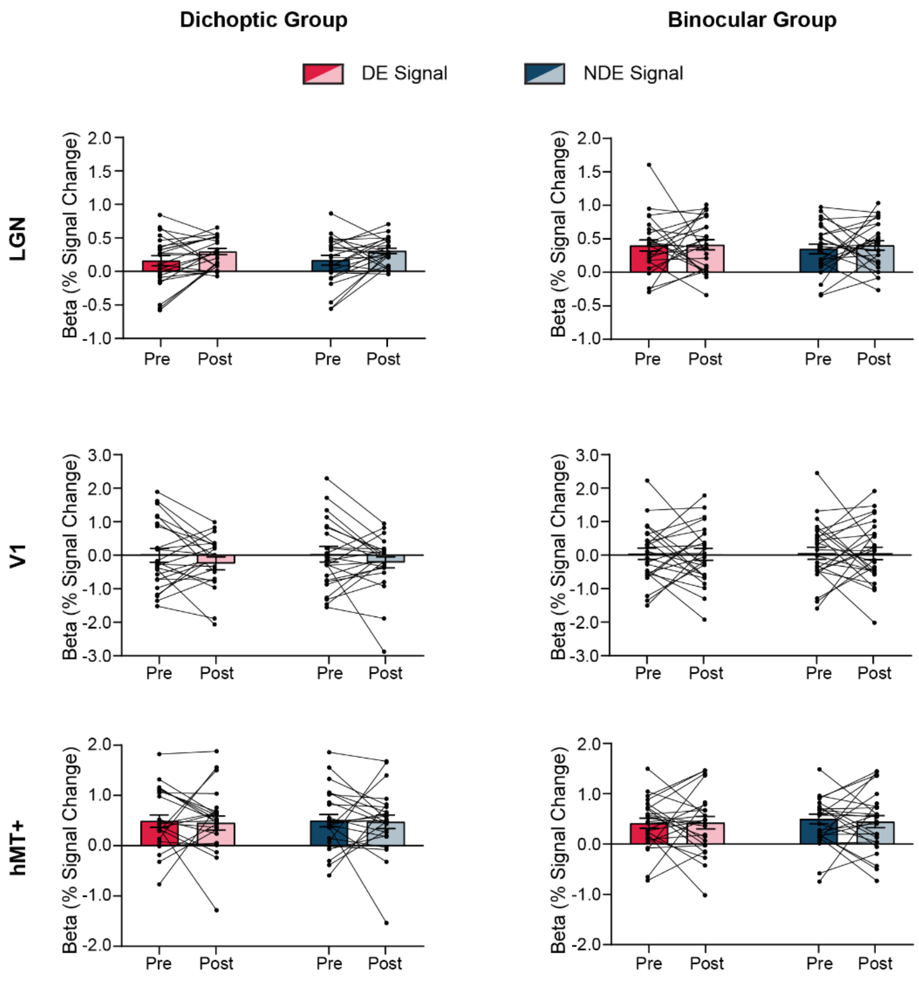

Supplementary Figure 4. GLM beta weights (% signal change) of the two stimulus configurations, i.e. [signal dots presented to the dominant eye (DE signal) and signal dots presented to the non-dominant eye (NDE signal)], presented independently for the three ROIs and the two training groups. Each connected dot pair in the figure corresponds to the GLM beta weights obtained from one participant before and after training. Error bars represent ±1 SEM.
